# Functions of cilia in cardiac development and disease

**DOI:** 10.1111/ahg.12534

**Published:** 2023-10-23

**Authors:** Wasay Mohiuddin Shaikh Qureshi, Kathryn E. Hentges

**Affiliations:** ^1^ Division of Evolution, Infection and Genomics, School of Biological Sciences, Faculty of Biology, Medicine, and Health, Manchester Academic Health Science Centre University of Manchester Manchester UK

**Keywords:** cardiac asymmetry, cardiac development, cilia, congenital heart defects, laterality

## Abstract

Errors in embryonic cardiac development are a leading cause of congenital heart defects (CHDs), including morphological abnormalities of the heart that are often detected after birth. In the past few decades, an emerging role for cilia in the pathogenesis of CHD has been identified, but this topic still largely remains an unexplored area. Mouse forward genetic screens and whole exome sequencing analysis of CHD patients have identified enrichment for *de novo* mutations in ciliary genes or non‐ciliary genes, which regulate cilia‐related pathways, linking cilia function to aberrant cardiac development. Key events in cardiac morphogenesis, including left–right asymmetric development of the heart, are dependent upon cilia function. Cilia dysfunction during left–right axis formation contributes to CHD as evidenced by the substantial proportion of heterotaxy patients displaying complex CHD. Cilia‐transduced signaling also regulates later events during heart development such as cardiac valve formation, outflow tract septation, ventricle development, and atrioventricular septa formation. In this review, we summarize the role of motile and non‐motile (primary cilia) in cardiac asymmetry establishment and later events during heart development.

## 1 INTRODUCTION

The heart is one of the first organs to form during embryogenesis, functioning to ensure that an adequate supply of nutrients and oxygen moves throughout the developing embryo (Buckingham et al., 2005; C. M. J. Tan & Lewandowski, 2020). Heart development is a complex process that requires precise spatiotemporal gene expression to orchestrate the accurate formation of a four‐chambered organ with a connected circulatory system (Bruneau, 2013; Buijtendijk et al., 2020; Houyel & Meilhac, 2021). Errors during these precisely controlled processes can lead to structural malformations known as congenital heart defects (CHDs; Althali & Hentges, 2022; Morton et al., 2022; Samsa et al., 2015). CHDs are present in nearly 1% of live births, thereby making CHDs the most common birth defect (Althali & Hentges, 2022; van der Linde et al., 2011). Several genetic and non‐genetic factors contribute to the development of CHD (S. S. Patel & Burns, 2013; Peng et al., 2019; Shiaulou Yuan et al., 2013). Understanding the etiology of CHD can improve diagnostic tools and help affected families to understand the severity of the disease (Dodge‐Khatami, 2016; Houyel & Meilhac, 2021; Pierpont et al., 2018). This knowledge is valuable because recent advances in CHD diagnosis and treatment have substantially increased the survival of CHD patients into adulthood (Mandalenakis et al., 2020).

In the past few decades, several studies have highlighted the importance of hair‐like cellular structures called cilia during cardiac development. Defects in cilia structure or function contribute to a variety of CHDs resulting from errors in cardiac asymmetry establishment, morphological arrangement, and valve development (Clement et al., 2009; Y. Li et al., 2015; X. Li et al., 2020; Slough et al., 2008; Toomer et al., 2019; Watanabe et al., 2003; Willaredt et al., 2012). In this review, we will summarize the current knowledge revealing how motile and non‐motile cilia at the left–right (L‐R) organizer (LRO) influence cardiac asymmetric morphogenesis and discuss the role of cardiac primary cilia in heart development and CHDs.

## 2 THE ARCHITECTURE OF CILIA

**FIGURE 1:**
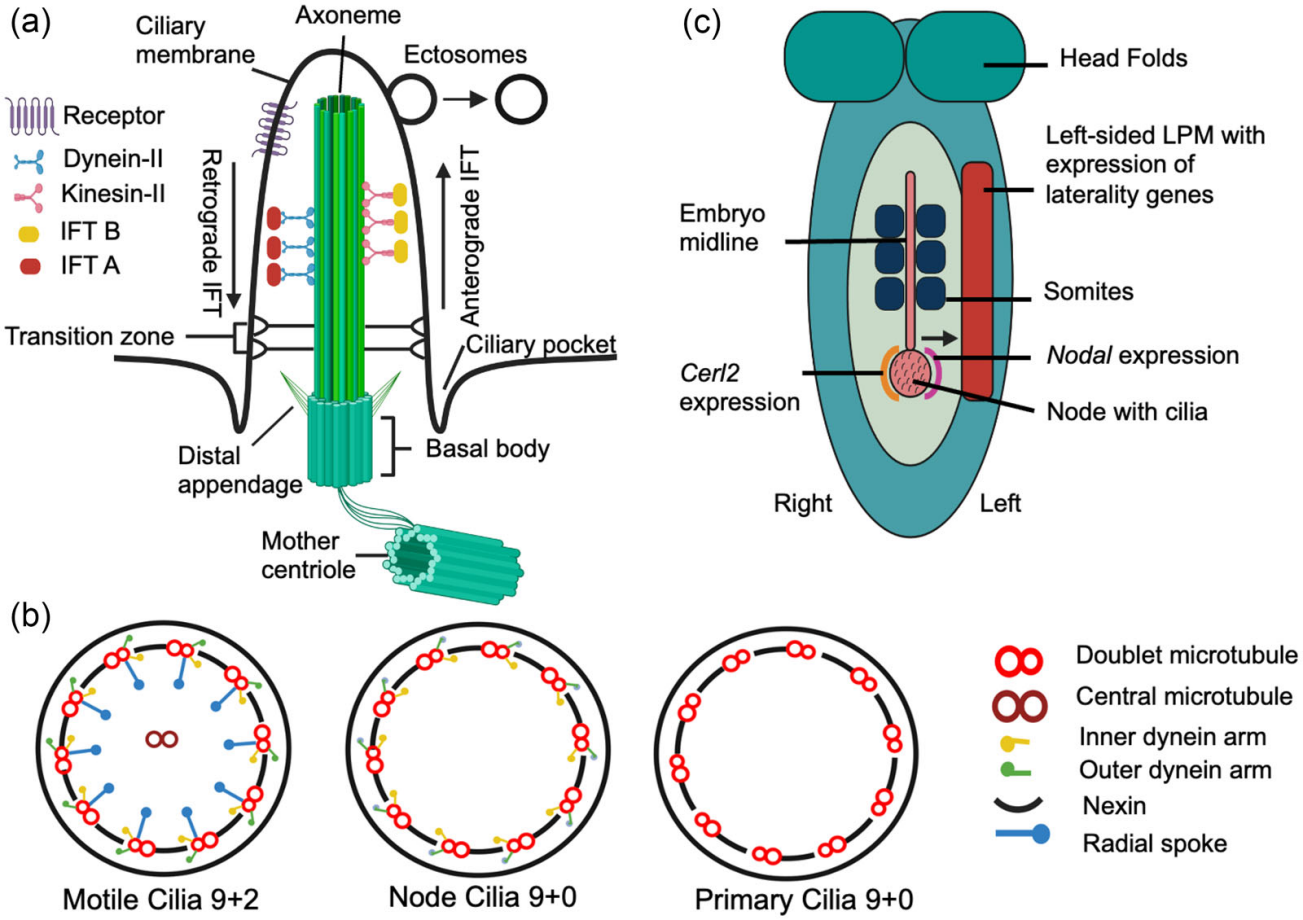
Mouse embryonic node, cilia structure, and doublet microtubule arrangements in motile and primary cilia. (a) Structurally, the cilium consists of a basal body, an axoneme, and dynein or kinesin proteins transporting the IFT complex proteins along the axoneme. (b) The axoneme microtubule arrangement. Motile cilium showing the 9 + 2 arrangement of microtubules along with inner dynein arms, outer dynein arms and radial spokes. The node motile cilia lack the central microtubule pair (9 + 0) and radial spokes. The primary cilia lack the motility components including the central microtubule pair (9 + 0). (c) Mouse embryo showing the cilia present at the embryonic node. The anticlockwise movement of motile cilia at the embryonic node generates leftward fluid flow and activates laterality gene expression and signaling on the left of the embryo in the lateral plate mesoderm. Figure created with Biorender.com and Microsoft Paints.

Cilia are evolutionarily conserved, unique, sensory antennae‐like structures of several micrometers in length that protrude from the apical surfaces of most vertebrate cells (Drummond, 2012; Long & Huang, 2019; Malicki & Johnson, 2017; Narasimhan & Roy, 2015; Satir & Christensen, 2007; Tasouri & Tucker, 2011; Willaredt et al., 2012). Cilia sense external environmental queues including light, low‐molecular‐weight chemicals, proteins, and mechanical stimuli and transfer the biochemical information to the cell to regulate cellular signaling pathways. Cilia also secrete ectosomes that signal to recipient cells to regulate cell– communication (Ferreira et al., 2019; Koefoed et al., 2014; Long & Huang, 2019; Malicki & Johnson, 2017; Toomer et al., 2019). Cilia function is critical for embryonic patterning, organogenesis, and adult tissue/organ homeostasis (Kim et al., 2023). During the mid‐20th century, the detailed structure of cilia was revealed using transmission electron microscopy (Fawcett & Porter, 1954; Gibbons, 1961; Rieder et al., 1979). Structurally, cilia have a nine parallel doublet microtubule‐based cytoskeleton core called the axoneme, surrounded by a ciliary membrane that is continuous with the cell membrane (Fawcett & Porter, 1954; Figure 1a). The ciliary axoneme is anchored at the base by the basal body, which is a modified centriole. The basal body is tethered to the plasma membrane via distinct appendages called transition fibers, establishing a selective gating system that controls protein trafficking between the cytoplasm and cilia (May et al., 2021; Miller et al., 2013; for more detail about cilia structure, see reviews Mill et al., 2023; Mirvis et al., 2018).

Depending upon their motility and axonemal architecture, cilia can be classified as either motile or non‐motile (primary cilia; Ishikawa, 2017; Kiesel et al., 2020; Sun et al., 2019). Motile cilia are found at the embryonic node (Alten et al., 2012), brain ventricles (Olstad et al., 2019), oviducts (Shuiqiao Yuan et al., 2021), and airway epithelium (Shah et al., 2009). The motile cilia axoneme usually has a 9 + 2 arrangement of microtubules, equipped with dynein arms and radial spokes, which generate beats via dynein arm‐driven adenosine triphosphate (ATP) hydrolysis, to produce fluid movement or propel gametes (Figure 1b; Hale & Sadoshima, 2022; Hjeij et al., 2014; Koefoed et al., 2014; Reiter & Leroux, 2017). However, node motile cilia are an exception because they lack the central microtubule pair and radial spokes (9 + 0 with dynein arms) but can generate rotational movement that results in fluid flow (Figure 1b; Huang et al., 2009; Ishikawa, 2017; Shinohara et al., 2015). Non‐motile or primary cilia have a 9 + 0 microtubule arrangement, missing the central microtubule pair, dynein arms, and radial spokes (Sun et al., 2019; Figure 1b). A single primary cilium protrudes from the surface of most vertebrate cell types. Primary cilia were previously considered as vestigial evolutionary remnants with no function (Toomer et al., 2019). However, primary cilia have been discovered to be dynamic structures that function as mechanosensors and signaling hubs that are fundamental to the development and maintenance of many tissues and organs (Fulmer et al., 2020; Miller et al., 2013; Tasouri & Tucker, 2011).

Recent findings have identified that cilia possess local protein translation machinery to maintain ultrastructure and function (Hao et al., 2021). However, for the transport of cytoplasmic proteins across the axoneme, cilia rely on the intraflagellar transport system (IFT). The IFT is a specialized evolutionarily conserved bidirectional microtubule motor‐based transport system, required for the growth and maintenance of both motile and primary cilia (Fry et al., 2014; Rosenbaum & Witman, 2002). The IFT complexes (IFT‐A retrograde transport and IFT‐B anterograde transport), along with kinesins, dynein motor proteins, and adapter molecules, ferry proteins and other cargo across the axoneme and in and out of the cilia (Koefoed et al., 2014; May et al., 2021; K. Patel & Smith, 2023; Reiter & Leroux, 2017; Tasouri & Tucker, 2011; Toomer et al., 2019; Willaredt et al., 2012; Figure 1). Mutations in the IFT complex affect the entire cilia transport system. Mutations in IFT‐B members result in short or absent cilia, while with IFT‐A, defects typically result in distorted cilia with a distended tip due to the accumulation of stranded IFT cargo (Pigino et al., 2009; Y. Zhang et al., 2018; for more detail about IFT system, see reviews Lechtreck, 2015; Taschner & Lorentzen, 2016).

Cilia possess complex repositories of channels and membrane‐spanning receptors that transduce mechanical, electrical, and chemical signals from the extracellular environment to the cytoplasm in a tissue‐specific and time‐dependent context (Christensen et al., 2007; Nishimura et al., 2018; Pala et al., 2017). The co‐localization of multiple channels and signaling components within the cilia raises the possibility that cilia coordinate the cross‐talk between signaling pathways (Nachury, 2014). These pathways include G protein‐coupled receptors (GPCRs; Brewer et al., 2023; Mykytyn & Askwith, 2017), receptor tyrosine kinase (Christensen et al., 2017), hedgehog (Hh; Bangs & Anderson, 2017; Tasouri & Tucker, 2011), Wingless‐related integration site (Wnt) (Kyun et al., 2020; Wallingford & Mitchell, 2011), platelet‐derived growth factor receptor (PDGFR; Schmid et al., 2018; Schneider et al., 2005), transforming growth factor‐beta (TGF‐β)/bone morphogenic protein (BMP; Álvarez‐Satta et al., 2021; Clement et al., 2013; Gencer et al., 2017), planar cell polarity (PCP; Borovina et al., 2010; Park et al., 2008; Ross et al., 2005; Song et al., 2010), and extracellular matrix (ECM; Battini et al., 2006; McGlashan et al., 2006). Of these cilia‐regulated pathways, several are known to be important for cardiac development (Gabriel et al., 2021). Defects in cilia structure and aberrant signaling via the cilium result in a variety of cilia‐associated diseases, collectively known as ciliopathies (Baker & Beales, 2009; Hale & Sadoshima, 2022; Long & Huang, 2019; Reiter & Leroux, 2017; Toomer et al., 2019). There are nearly 200 distinct ciliopathies (Rao Damerla et al., 2014). First‐order ciliopathies are caused by ciliary gene disruption, while second‐order ciliopathies result from defects in non‐ciliary genes required for cilia function (Reiter & Leroux, 2017; for more detail regarding first‐order and second‐order cilia genes and associated ciliopathies, see Vasquez et al., 2021).

## 3 LRO: THE BODY'S SYMMETRY BREAKER

Vertebrate visceral organs and their associated vasculature are characteristically arranged asymmetrically inside the body in an orientation known as situs solitus (Fujinaga, 1997; Nöthe‐Menchen et al., 2019; Sampaio et al., 2014; Shapiro et al., 2014; Stevens et al., 2010; Watanabe et al., 2003). Organ asymmetry defects and associated diseases can cause life‐threatening complications (Saba et al., 2022). Failure to establish the characteristic internal organ asymmetry can lead to laterality defects such as heterotaxy (random orientation of organs) or situs inversus (mirror image reversal of organs; Chen et al., 2019; Eitler et al., 2022; Saba et al., 2022). In several vertebrates, bilateral symmetry is broken at a transient embryonic structure known as the LRO (Blum et al., 2007). The LRO name varies between species; in mice, it is called the node (Nonaka et al., 1998, 2002; Figure 1c), in frog, it is the gastrocoel roof plate (Sáenz‐Ponce et al., 2012; Schweickert et al., 2007), while in zebrafish, it is the Kupffer's vesicle (KV; Kramer‐Zucker et al., 2005). The motile cilia at embryonic LRO generate extracellular fluid flow (nodal flow), causing the expression of asymmetric genes in the lateral plate mesoderm (LPM) and activation of downstream signaling effectors, which co‐ordinates the asymmetric morphogenesis of the internal organs (Hirokawa et al., 2012; Kawasumi et al., 2011; Larkins et al., 2012; Watanabe et al., 2003).

The mouse embryonic node is a tear‐shaped, mesodermal‐derived, epithelial pit at the posterior end of the notochord containing monociliated cells (Hirokawa et al., 2009; Sulik et al., 1994; Yamanaka et al., 2007). The cilia on the node cells are tilted posteriorly, which produces leftward flow when the cilia are motile (Nonaka et al., 2005). Defects in PCP pathways, which regulate the tilt direction of node motile cilia, result in laterality defects (Mahaffey et al., 2013; Song et al., 2010). The motile cilia at the embryonic node beat radially in an anticlockwise direction to perpetuate a leftward flow, while non‐motile or sensory cilia, which extend from the crown cells surrounding the pit, sense the flow (McGrath et al., 2003; Yoshiba et al., 2012). This flow sensing triggers a conserved molecular cascade by initiating laterality gene expression in the LPM, which subsequently culminates in the correct positioning of the visceral organs (McGrath et al., 2003; Nonaka et al., 2002; Sampaio et al., 2014). In mouse, a total of nearly 200–300 motile cilia at the embryonic node generate nodal flow; however, as few as two motile cilia are enough to break embryonic internal bilateral symmetry (Shinohara et al., 2012).

At the molecular level, embryonic leftward fluid flow activates the nodal signaling cascade (Brennan et al., 2002). *Nodal*, a TGF‐β family signaling molecule, is asymmetrically expressed at the node and activates left–right patterning gene expression in the LPM (Figure 1c; Collignon et al., 1996; Norris & Robertson, 1999; Saijoh et al., 2003; Sakuma et al., 2002). *Nodal* asymmetrical expression is restricted to the left side of the node by the *Nodal* antagonist *Cerl2*. *Cerl2* is initially expressed bilaterality around the node at the early headfold stage; however, leftward nodal flow initiates *Cerl2* mRNA decay on the left side, which is further enhanced by a *Wnt3–Cerl2* interlinked feedback loop (Belo et al., 2017; Inácio et al., 2013; Marques et al., 2004; Nakamura et al., 2012; Schweickert et al., 2010). *Nodal* activity on the left side of the node triggers the initiation of the laterality gene expression program (Brennan et al., 2002; Norris et al., 2002). Cells that receive nodal signaling adopt a left‐sided cell fate (Yamamoto et al., 2003). *Nodal* expression is further maintained by the downstream transcription factors *lefty* and *Pitx2* (Adachi et al., 1999; Logan et al., 1998; Oki et al., 2009; Piedra et al., 1998; Yoshioka et al., 1998). Once *Nodal* expression has ceased, asymmetric *Pitx2* expression in the heart, foregut, cardinal vein, and umbilical vein is maintained by the transcription factor *Nkx2‐5* (Shiratori et al., 2001). These findings suggest that node cilia‐induced laterality gene expression and activation of downstream signaling play a decisive role in asymmetrical development and patterning of the internal organs including the heart.

## 4 PRESENCE OF CILIA IN CARDIAC DEVELOPMENT

**FIGURE 2:**
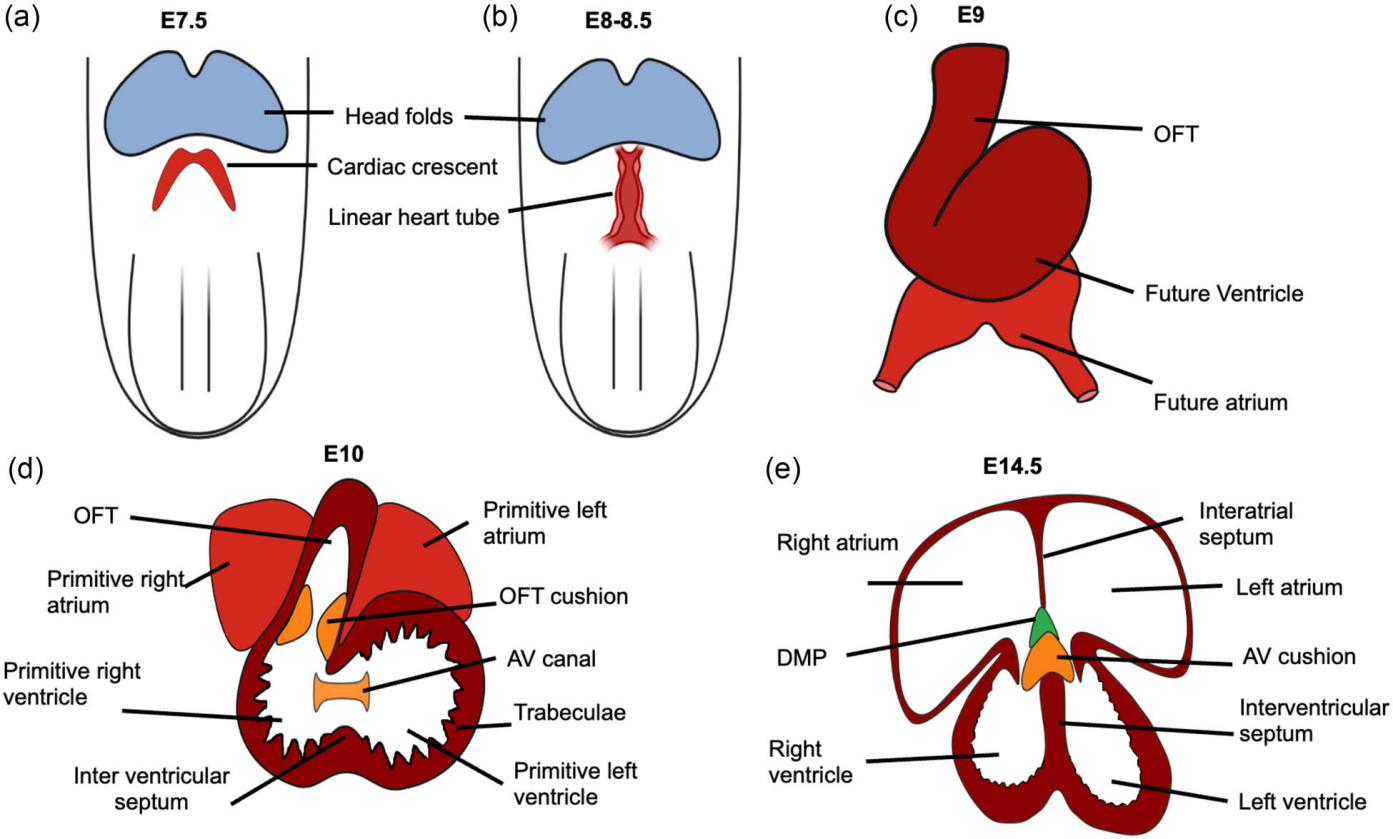
The stages of mouse heart development. (a) Mouse heart development at Embryonic Day 7.5, when cardiac progenitor cells migrate toward the midline to form a crescent‐shaped heart field. (b) The crescent‐shaped heart field cells converge at the midline to form a linear heart tube. (c) The linear heart tube undergoes dextral looping to shape the future four‐chambered heart. (d) The ventricular wall of the looped heart undergoes trabeculation, and endocardial cushion cells protrude into the heart tube to start to form the cardiac valves. (e) The four‐chambered heart is formed around E14.5 with the presence of atrial and ventricular septa. AV, atrioventricular; DMP, dorsal mesenchymal protrusion; OFT, outflow tract. Figure created with Biorender.com and Microsoft Paints.

Cardiac organogenesis in vertebrates is a highly complex, tightly coordinated series of gene expression and signaling events that require the migration of different types of progenitor cells to form the heart (Koefoed et al., 2014; Münsterberg & Yue, 2008). The earliest known cardiac progenitor cells express the transcription factor *Mesp1* and are derived from mesodermal cells present in the anterior primitive streak region (Kitajima et al., 2000; Saga et al., 1996, 1999). These cardiac progenitor cells from the anterior primitive streak region migrate under the embryonic headfolds and form a bilaterally symmetrical crescent‐shaped heart field at the midline (Figure 2a; Ivanovitch et al., 2017; Kuhn & Wu, 2010). In the mouse, the heart starts to form after E7.5, when cells from the two crescent‐shaped heart fields migrate toward the ventral midline and converge to form a linear heart tube (Figure 2b; Kelly, 2012; Willaredt et al., 2012; M. Wu, 2018). The linear heart tube undergoes dextral looping to form an asymmetric four‐chambered heart (Figure 2c). The cardiac looping process is controlled by several factors including nodal signaling at the LRO, which specifies left identity in myocardial precursor cells (Desgrange et al., 2020).

Once the linear heart tube loops, the endocardial cushions (cardiac valve precursor structures located at the atrioventricular [AV] junction and within the outflow tract [OFT]) start to swell. These precursor cells then undergo epithelial‐to‐mesenchymal transition (EMT) and secrete ECM resulting in the formation of the heart valves (O'Donnell & Yutzey, 2020; Person et al., 2005; Figure 2d). Additionally, following looping, the heart wall forms a network of luminal projections known as trabeculae. The presence of cardiac trabeculae increases cardiac output and facilitates nutrient and oxygen exchange in the embryonic myocardium prior to the establishment of the coronary vasculature. After the establishment of coronary vasculature, the trabeculae undergo extensive remodeling and coalesce together with the cardiac wall to form a thick muscular layer (Lai et al., 2010; Qu et al., 2022; Samsa et al., 2013, 2015; M. Wu, 2018; Figure 2e).

Two extra cardiac cell sources, the cardiac neural crest cells (Schleiffarth et al., 2007; Stefanovic et al., 2021) and epicardial cells (J. Li et al., 2017; Ridge et al., 2017) also contribute to the development of the heart. The cardiac neural crest cells migrate from the hindbrain region including rhombomeres 6‐8 and contribute to the development of the OFT, cardiac valves, cardiac septa, and arteries of the heart (George et al., 2020; Hutson & Kirby, 2007; Schleiffarth et al., 2007; Stefanovic et al., 2021; Yan et al., 2021). In the mouse, around E10.5, the cardiac OFT then undergoes septation to form the aorta and pulmonary artery, which are required to carry oxygenated and deoxygenated blood in and out of the heart (Schleiffarth et al., 2007; Stefanovic et al., 2021). Epicardial cells migrate from the pro‐epicardial organ (located at the venous pole of the heart) and attach to the surface of heart around E9.5 in the mouse. These cells underdo EMT and contribute to different cell types in the heart, facilitating establishment of the coronary circulation (J. Li et al., 2017; Ridge et al., 2017; see fig. 3 in the review from Clowes et al., 2014). The process of four‐chambered heart formation is completed around E14.5 in mouse embryos (Savolainen et al., 2009).

Primary cilia have been reported to appear throughout the embryonic heart development (Slough et al., 2008; Willaredt et al., 2012). Ultrastructural analysis of cardiac cilia using transmission electron microscopy shows the presence of cilia with nine pairs of microtubules, lacking the central microtubule pair and cilia motility machinery, confirming the presence of primary cilia (Slough et al., 2008; Toomer et al., 2019; Willaredt et al., 2012). Slough et al. (2008) illustrated the presence of primary cilia on mouse embryonic heart cells as early as E9.5 of development (Slough et al., 2008). During cardiac development, cilia are found on all cell types in the heart including cardiomyocytes, endocardial cells, epicardial cells, and cardiac cushion cells (Gerhardt et al., 2013; Myklebust et al., 1977; Slough et al., 2008). However, using the cilia cytoskeleton marker acetylated α‐tubulin and the basal body marker pericentrin, Gerhardt et al. (2013) observed a lack of cilia on ventricular septa and ventricular cells close to the septum between E10.5 and E12.5 of heart development. Cilia on cardiac cushion mesenchymal cells are present in a ciliary pocket of varying depth with a random orientation. Endocardial cell primary cilia are orientated toward the lumen of the inflow and OFTs, while atrial and ventricular primary cilia are oriented toward the blood‐filled cardiac chambers, and epicardium primary cilia are oriented toward the pericardial space (Diguet et al., 2015; Slough et al., 2008; Willaredt et al., 2012). The presence and length of cilia in the mitral valves correlate with the type of ECM produced during heart development. For example, in early development, the valves have abundant proteoglycans but little collagen and express cilia on nearly all cells. As development progresses, by Postnatal Day 0, there is an increase in collagen expression in the valves concomitant with a reduction in the number and length of primary cilia. These cilia are mainly localized to regions with low collagen expression (Toomer et al., 2019).

A growing body of work has also identified the importance of primary cilia in cardiac development (Burnicka‐Turek et al., 2016; Fulmer et al., 2019; Gerhardt et al., 2013; Hartill et al., 2018; Y. Li et al., 2015; Slough et al., 2008; Willaredt et al., 2012). Using a forward genetic screen in the mouse, Y. Li et al. (2015) identified 61 genes, in which pathogenic mutations could produce echocardiographically identifiable CHDs. Of these genes, 35 genes encoded either motile or primary cilia proteins, with many of the remaining genes being involved in cilia‐related signaling (16 genes) or vesicular trafficking (10 genes), which are required for cilia formation and function (Y. Li et al., 2015).

## 5 LRO CILIA AND CARDIAC ASYMMETRY

Cilia function at the LRO is critical for the establishment of cardiac left–right (L‐R) asymmetry. Both motile and sensory cilia (primary cilia) at the mouse embryonic node are indispensable for the establishment of left–right asymmetry (McGrath et al., 2003; Nonaka et al., 1998; Yoshiba et al., 2012), which is important for the early stages of heart morphogenesis and appropriate connections to the vasculature (Koefoed et al., 2014). In mice, the first morphological sign of left–right asymmetry is the dynamic right‐sided looping of the primitive heart tube, followed by embryonic turning that converts the embryo from a lordotic to a fetal position (Chatterjee et al., 2007; Honda et al., 2020). Heart asymmetry is critical for efficient oxygenation of the blood and establishment of the systemic and pulmonary circulation (Francis et al., 2012). Defects in laterality establishment significantly increase the risk of CHD. Nearly 3% of all CHD results from heterotaxy, and complex CHD is often associated with heterotaxy (57% in heterotaxy patients vs. 1% in the general population; Agarwal et al., 2021; Burnicka‐Turek et al., 2016; Djenoune et al., 2022; Merklin & Varano, 1963; Slough et al., 2008). Here, we will discuss some of the known LRO‐related cilia components that regulate cardiac asymmetry establishment.

### 5.1 Nodal cilia motility component defects and cardiac asymmetry

The primary cilia dyskinesias (PCDs; OMIM 244400) are a set of diseases associated with motile cilia dysfunction (Y. Li et al., 2016; Reiter & Leroux, 2017). PCD is often associated with situs inversus, heterotaxy, and complex CHD (Best et al., 2019; Francis et al., 2012; Harrison et al., 2016). Y. Li et al. (2015) reported that most PCD‐causing motile cilia genes are also known to cause CHD. The dysfunction of the motile cilia outer dynein arms (ODA), which generate motor force for ciliary beating (Zimmermann et al., 2023), is the major cause of PCD (Wallmeier et al., 2016). ODA genes including *Dnaic1* (Francis et al., 2012) (see Table 1 for the list of cilia genes mentioned in this review), *DNAH5* (Ibañez‐Tallon et al., 2002; Nöthe‐Menchen et al., 2019; S. Y. Tan et al., 2007), *DNAH11* (S. Liu et al., 2019; Xia et al., 2021), or ODA‐docking genes like *ARMC4* (Hjeij et al., 2013; Onoufriadis et al., 2014), *TTC25* (Wallmeier et al., 2016), *Ccdc39* (Solomon et al., 2017), *DNAH10* (C. Liu et al., 2018), and *MNS1* (Ta‐Shma et al., 2018) are frequently associated with motile cilia dysfunction, left–right patterning defects,, and cardiac asymmetry defects. Hjeij et al. (2014) have identified a mutation in the ODA docking gene *CCDC151* in PCD individuals with dextrocardia. *CCDC151* mutant cilia fail to assemble with the ODA component DNAH5 and the ODA‐docking complex. This altered complex formation results in cilia structural changes with complete loss of ODA and impaired ciliary beating, leading to a spectrum of situs defects associated with complex heart defects (Hjeij et al., 2014). Mutations in inner dynein arm (IDA) genes including *DNAH6* (Y. Li et al., 2016) or *DNAH7* (Y. Z. Zhang et al., 2002) impair the formation of the IDA structure and central microtubule pair, which are required for cilia motility. Knockdown of *dnah6* in zebrafish embryos causes a constellation of heterotaxy phenotypes with reduced cilia length at the KV, disruption of left‐sided southpaw (zebrafish Nodal homolog) expression, abnormal body curvature, and altered orientation of heart and gut looping (Y. Li et al., 2016). Likewise, disruption of the coiled‐coil domain containing‐40 (*Ccdc40*) gene, which regulates the assembly of the IDA and the dynein regulatory complexes (Becker‐Heck et al., 2011), drastically reduces the cilia length at the mouse node, affecting cilia motility and compromising nodal flow, resulting in situs inversus or heterotaxia with cardiac looping defects (Becker‐Heck et al., 2011; Sugrue & Zohn, 2017).

**TABLE 1:** List of cilia structure, function, and signaling genes (mentioned in the article), in alphabetical order, that cause cardiac defects when mutated.

Gene symbol	Gene name	First/second‐order cilia gene	Mutant phenotype	Reference
*ARMC4*	Armadillo repeat containing 4	First order	Laterality defects, cardiac looping defects	(Hjeij et al., 2013; Onoufriadis et al., 2014)
*CCDC 40*	Coiled‐coil domain containing 40	First order	Laterality defects, cardiac looping defects	(Becker‐Heck et al., 2011; Sugrue & Zohn, 2017)
*CCDC151*	Coiled‐coil domain containing 151	First order	Laterality defects, dextrocardia, ventricular septal defects	(Hjeij et al., 2014)
*CCDC39*	Coiled‐coil domain containing 39	First order	Laterality defects, cardiac looping defects	(Solomon et al., 2017)
*CRELD1*	Cysteine Rich With EGF Like Domains 1	Second order	Atrioventricular septal defects (AVSDs), valve defects	(Beckert et al., 2021, Burnicka‐Turek et al., 2016)
*DHH*	Desert hedgehog	Second order	Mitral valve prolapse (MVP)	(Fulmer et al., 2020)
*DNAAF1*	Dynein axonemal assembly factor 1	First order	Laterality defects, cardiac looping defects	(Hartill et al., 2018)
*DNAAF3*	Dynein axonemal assembly factor 3	First order	Laterality defects, cardiac looping defects	(Mitchison et al., 2012)
*DNAH5*	Dynein axonemal heavy chain 5	First order	Laterality defects, cardiac looping defects	(Nöthe‐Menchen et al., 2019)
*DNAH6*	Dynein axonemal heavy chain 6	First order	Laterality defects, cardiac looping defects	(Y. Li et al., 2016)
*DNAH7*	Dynein axonemal heavy chain 7	First order	Laterality defects, cardiac looping defects	(Y. J. Zhang et al., 2002)
*DNAH 10*	Dynein axonemal heavy chain 10	First order	Laterality defects, cardiac looping defects	(C. Liu et al., 2018)
*DNAH11*	Dynein axonemal heavy chain 11	First order	Laterality defects, AVSDs	(Bartoloni et al., 2002; Burnicka‐Turek et al., 2016; Dougherty et al., 2016; S. Liu et al., 2019; Xia et al., 2021)
*DNAIC1*	Dynein Axonemal Intermediate Chain 1	First order	Laterality defects, cardiac looping defects	(Francis et al., 2012)
*DYX1C1(DNAAF4)*	Dyslexia Susceptibility 1 Candidate 1	First order	Laterality defects, cardiac looping defects	(Tarkar et al., 2013)
*DZIP1*	DAZ interacting zinc finger protein 1	First order	MVP	(Toomer et al., 2019)
*EXOC5*	Exocyst complex component 5	First order	Bicuspid aortic valve disease and aortic stenosis	(Fulmer et al., 2019)
*FTM (Rpgrip1l)*	Fantom	First order	Laterality defects, cardiac looping defects, AVSD	(Gerhardt et al., 2013; Vierkotten et al., 2007)
*GPR22*	G protein‐coupled receptor (GPCR) 22	First order	Laterality defects, cardiac looping defects, cardiac edema	(Verleyen et al., 2014)
*GRK5*	GPCR kinase 5	Second order	Laterality defects, cardiac looping defects, valve development defects	(Burkhalter et al., 2013; Casar Tena et al., 2015)
*IFT20*	Intraflagellar transport 20	First order	Proepicardial organ and myocardial tissue size defects	(Peralta et al., 2020)
*IFT46*	Intraflagellar transport 46	First order	Laterality defects, cardiac looping defects	(Lee et al., 2015)
*IFT54 (TRAF3IP1)*	Intraflagellar transport 54(TRAF3 interacting protein 1)	First order	Proepicardial organ and myocardial tissue size defects	(Peralta et al., 2020)
*IFT57*	Intraflagellar transport 57	First order	Laterality defects, cardiac looping defects	(Houde et al., 2006)
*IFT74*	Intraflagellar transport 74	First order	Laterality defects, cardiac looping defects, AVSD, hypoplastic left heart	(Bakey et al., 2023)
*IFT88*	Intraflagellar transport 88	First order	Laterality defects, cardiac looping defects, outflow tract defects, ventricular trabeculation defects, cardiac cushion EMT defects, valves defects, proepicardial organ and myocardial tissue size defects	(Burns et al., 2019; Clement et al., 2009; Murcia et al., 2000; Peralta et al., 2020; Toomer et al., 2019; Willaredt et al., 2012)
*IFT172*	Intraflagellar transport 172	First order	Laterality defects, cardiac looping defects	(Gorivodsky et al., 2009)
*INVS*	Inversin	First order	Laterality defects, cardiac looping defects	(Lowe et al., 1996, Okada et al., 1999; Watanabe et al., 2003; Yokoyama et al., 1993)
*KIF3A*	kinesin family member 3A	First order	Laterality defects, cardiac looping defects	(Takeda et al., 1999)
*KIF3B*	kinesin family member 3B	First order	Laterality defects, cardiac looping defects	(Nonaka et al., 1998)
*MEGF8*	Multiple Epidermal Growth Factor‐like Domains 8	Second order	Laterality defects, cardiac looping defects	(Y. Li et al., 2015; Z. Zhang et al., 2009)
*MKS1*	MKS Transition Zone Complex Subunit 1	First order	AVSDs	(Burnicka‐Turek et al., 2016; Cui et al., 2011)
*MNS1*	Meiosis specific nuclear structural 1	First order	Laterality defects	(Ta‐Shma et al., 2018)
*PDGFR‐α*	Platelet‐derived growth factor receptor‐alpha	Second order	MVP	(Moore et al., 2021)
*PKD1*	Polycystin 1, transient receptor potential channel interacting	First order	AVSDs, myocardial wall thinning, double‐outlet right ventricle, cardiac valves defects	(Boulter et al., 2001; Juan et al., 2023)
*PKD1L1*	Polycystin 1 like 1, transient receptor potential channel interacting	First order	Laterality defects, cardiac looping defects, cardiac valve defects	(Field et al., 2011; Juan et al., 2023)
*PKD2*	Polycystin 2, transient receptor potential cation channel	First order	Laterality defects, cardiac looping defects, cardiac septation defects, cardiac valve defects	(Juan et al., 2023; Pennekamp et al., 2002; G. Wu et al., 2000)
*TBC1D32*	TBC1 domain family member 32	First order	Laterality defects, cardiac looping defects	(Y. Li et al., 2015)
*TCTN2*	Tectonic family member 2	First order	Ventricular septal defects	(Sang et al., 2011)
*TTC25*	Tetratricopeptide repeat domain 25	First order	Laterality defects, cardiac looping defects	(Wallmeier et al., 2016)

*Note*: First‐order genes are those that encode proteins required to form the cilia, cilia motility, or for cilia cargo transport. Second‐order genes are those that participate in signaling at the cilium or have undefined roles in the cilium.

Several other cilia genes like *DNAAF1* (Hartill et al., 2018), *DNAAF3* (Mitchison et al., 2012), and *DYX1C1(DNAAF4)* (Tarkar et al., 2013), which are required for dynein heavy chain assembly and cilia motility, also display PCD with heterotaxy and complex CHD when mutated. Mutations in the central microtubule pair and radial spoke genes are often associated with PCD but not heterotaxy (Best et al., 2019), as node motile cilia lack these structures (Figure 1b).

### 5.2 Nodal cilia non‐motility component defects and cardiac asymmetry

The non‐motile structural components of cilia such as centrosomal proteins, IFTs, and transition zone components also affect cardiac left–right asymmetry establishment (Gorivodsky et al., 2009; Houde et al., 2006; Lee et al., 2015; Murcia et al., 2000; Shylo et al., 2020; C. Wu et al., 2014). Takeda et al. (1999) and Nonaka et al. (1998) showed that cilia at the node are indispensable for breaking bilateral symmetry by deleting the Kinesin superfamily proteins Kif3a (Takeda et al., 1999) and Kif3b (Nonaka et al., 1998), respectively, resulting in loss of node cilia, symmetry defects, and randomization of cardiac looping (Marszalek et al., 1999; McGrath et al., 2003; Nonaka et al., 1998; Takeda et al., 1999). The restoration of *Kif3a* expression only in crown cells (which possess sensory cilia) in *Kif3a* mutant embryos allows a response to artificially induced fluid flow and rescues *Nodal* and *Pitx2c* expression in the LPM. However, the rescue of cardiac looping defects was not analyzed (Yoshiba et al., 2012). Mice embryos with a faulty IFT system, such as mutants for the IFT complex B gene *Ift46*, die around E10–E10.5, with neural tube and heart defects. Pericardial edema and cardiac looping defects were observed, as well as a lack of cilia, at the embryonic node. The absence of nodal flow in these mutants results in bilateral *Lefty1* expression in the LPM and cardiac looping defects (Lee et al., 2015). Other IFT genes like *Ift57* (Houde et al., 2006), *Ift88* (Murcia et al., 2000), *Ift172* (Gorivodsky et al., 2009), and *Ift74* (Bakey et al., 2023) also showed defects in node cilia structure and function with randomized heart looping in homozygous mutants.

Several studies where cilia genes were genetically manipulated revealed normal but immobile node cilia with abnormal left–right cardiac asymmetry (Slough et al., 2008). Mice mutant for the protein *Inversin*, which localizes at the proximal end of the cilium near the basal body, show no obvious defects in node monocilia but have disrupted nodal flow and right‐sided *Nodal* expression in the LPM. *Inversin* mutant mice consistently display situs inversus with heart looping defects (Lowe et al., 1996; Okada et al., 1999; Watanabe et al., 2003; Yokoyama et al., 1993).

### 5.3 Defects in genes associated with cilia signaling function and cardiac asymmetry

Several genes that regulate signaling pathways that function at the cilium have been identified as having a role in the establishment of cardiac asymmetry (Y. Li et al., 2015). The fluid flow at the node induces movement of the primary cilia on crown cells, provoking an increase in calcium concentration in the crown cells. This change in calcium concentration stimulates the nodal signaling cascade on the left side of the node, which is translated into activation of laterality gene expression (McGrath et al., 2003; Pennekamp et al., 2002). Disruption of the calcium gradient across the node results in ambiguous expression of Nodal pathway genes, generating symmetry in L‐R development (Takao et al., 2013; Shiaulou Yuan et al., 2015), and cardiac looping defects (Pennekamp et al., 2002). The calcium‐permeable channel Polycystin‐2 (*Pkd2)* and its binding partner Pkd1l1 form a complex, which localizes to all node cilia (Field et al., 2011; Kamura et al., 2011; Yoshiba & Hamada, 2014). This complex has mechanosensing properties and plays a critical role in calcium gradient establishment and activation of nodal signaling in the LPM (Field et al., 2011; Kamura et al., 2011; McGrath et al., 2003; Pennekamp et al., 2002; Schottenfeld et al., 2007). *Pkd2* (Kamura et al., 2011; Pennekamp et al., 2002) or *Pkd1l1* (Field et al., 2011) mutant embryos show laterality defects with abnormal heart looping, and altered embryonic turning, but no structural or functional defects in node motile cilia. The specific restoration of *Pkd2* in perinodal crown cells rescues the symmetry defects in *Pkd2* mutants (Yoshiba et al., 2012). The application of mechanical forces to immotile cilia at the LRO triggers intraciliary calcium ion transients (Djenoune et al., 2023; Katoh et al., 2023), confirming the mechanosensory role of non‐motile cilia at the LRO in the establishment of left–right asymmetry.

The Hh pathway is known to play an important role in cilia signaling (Goetz & Anderson, 2010). In a screen for CHD phenotypes, an enrichment in genes associated with Hh signaling was reported (Y. Li et al., 2015). Hh pathway‐associated genes begin to be expressed during the early stages of laterality establishment (Hu et al., 2017) and are required for heart tube asymmetry (Tsiairis & McMahon, 2009; X. M. Zhang et al., 2001). The negative regulators of Hh signaling, *Tbc1d32* and *Megf8*, cause heterotaxy with CHD in mouse mutants (Y. Li et al., 2015). Hh signaling‐induced heart looping abnormalities are associated with defects in myocardial differentiation and the failure to upregulate expression of the cardiac transcription factor *Nkx2.5* (X. M. Zhang et al., 2001). The knockdown or overexpression of the GPCR *gpr22* in zebrafish also results in changes in cilia length and structure with defective L‐R pattering and randomized cardiac looping leading to heart edema (Verleyen et al., 2014). Dysregulation of the mammalian target of rampamycin (mTOR) signaling pathway results in altered nodal cilia length, with symmetry defects (Burkhalter et al., 2019; Shiaulou Yuan et al., 2012). Knockdown of *grk5* in zebrafish augments mTORC1 signaling and fails to break the cardiac symmetry (Burkhalter et al., 2013; Casar Tena et al., 2015), also affecting the expression of genes in the heart, which are important for valve development (Burkhalter et al., 2013).

## 6 PRIMARY CILIA, HEART DEVELOPMENT, AND CHDs

Research on primary cilia has been ongoing since the 1960s, when primary cilia were initially distinguished as structurally different from motile cilia and found to exist in the majority of mammalian cells (Myklebust et al., 1977). Primary cilia play a crucial role in cell differentiation and embryonic development (May et al., 2021). Disorders linked to primary cilia have been identified across various organ systems (Hale & Sadoshima, 2022). Our understanding of the role of primary cilia in heart development is still in its infancy, but there is an increasing recognition of primary cilia as important biomechanical and molecular regulators of cardiac development (Toomer et al., 2019).

### 6.1 Primary cilia and associated signaling defects in cardiac valve development

Cilia‐regulated signaling and responses to changes in shear stress are important for cardiac valve development (Gabriel et al., 2021). Unlike other cilia‐related CHDs, valve diseases are more frequently identified in adults than in infants. However, the early signs of disease can be observed during cardiac development with changes in the valve ECM (Fulmer et al., 2020; Morningstar et al., 2021; Toomer et al., 2019). Bicuspid aortic valve (BAV) and mitral valve prolapse (MVP) are cardiac valve defects (LaHaye et al., 2014) commonly identified in syndromic diseases associated with cilia defects (Karp et al., 2012). Cardiac valve diseases often include complications such as cardiac arrhythmias, heart failure, and sudden cardiac death, which sometimes require surgical intervention (Coutsoumbas & Di Pasquale, 2021). Previous studies have shown that genetic ablation of cilia‐related genes disturbs cardiac valve ECM expression causing highly penetrant myxomatous phenotypes like BAV (Fulmer et al., 2019; Toomer et al., 2017) and MVP (Toomer et al., 2019). Primary cilia regulate aortic valve development by directly or indirectly altering the production of critical ECM components (Toomer et al., 2017). A genome‐wide association study (GWAS) using a cohort of BAV and control patients identified single nucleotide polymorphisms in several exocyst complex genes (*EXOC4, EXOC6, EXOC8*) that are important in regulating ciliogenesis through cargo shuttling to the membrane (Fulmer et al., 2019). The authors further verified the role of the exocyst complex in BAV by knocking down a key linker protein, Exoc5, in both mouse and zebrafish, resulting in a ciliogenesis defect with a BAV phenotype (Fulmer et al., 2019).

Recent studies have identified the role of primary cilia in MVP (Fulmer et al., 2020; Toomer et al., 2019). MVP is characterized by the mechanical incompetence of mitral valve leaflets with increased proteoglycan production and collagen and elastin fragmentation (Fry et al., 2014; Fulmer et al., 2020; Morningstar et al., 2021; Toomer et al., 2019). The known MVP‐causal genes *DCHS1* (Durst et al., 2015) and *FLNA* (Kyndt et al., 2007) also show reduced cilia length in the mitral valve in knockout models, confirming a role for cilia in MVP (Toomer et al., 2019). Toomer et al. (2019) conditionally deleted the ciliary IFT‐B gene *Ift88* using an endocardial cell‐specific *NfatC1^Cre^*. This results in loss of the cilia axoneme from the endocardial cell‐derived valve mesenchyme, yielding significantly decreased valve interstitial cell density. Additionally, there was robust activation of ECM gene pathways in the anterior mitral leaflets, an indicator of early‐stage myxomatous degeneration resulting in adult myxomatous valve pathology (Toomer et al., 2019). GWAS of MVP cases have identified significant enrichment of MVP‐associated variants in cilia genes. Whole exome sequencing of MVP patients has identified variants in *DZIP1* (Toomer et al., 2019). *DZIP1* is a cilia‐related gene, which regulates ciliogenesis or Hh signaling (Lapart et al., 2019; Wang et al., 2013; B. Zhang et al., 2015). Knock‐in mice of a human *DZIP1* mutation proved to be a genetically accurate model for non‐syndromic MVP with adult myxomatous mitral valves and functional MVP with a reduction in cilia length. At the transcriptome level, the comparison between human *DZIP1* mutation knock‐in mice and endocardial cell‐specific *Ift88* deletion showed similar ECM pathway changes (Toomer et al., 2019).

The cilia‐regulated pathway ligand, desert Hh (*dhh*), which is expressed within the cardiac valves, regulates cytoskeleton organization during valve leaflet development. The dhh signal originates from the endocardium, resulting in the paracrine cross‐talk between the endocardium and ciliated valve interstitial cells to shape the valve formation. Conditional deletion of *dhh* using either *NfatC1^enCre^* (specifically expressed in valve endocardial cells that do not undergo EMT) or *Tie2Cre* (expressed in endothelial or endocardial cells) resulted in an MVP phenotype with no change in cilia length, confirming endocardial cilia‐mediated paracrine cross‐talk in valve development (Fulmer et al., 2020). Conditional deletion of *PDGFRα* receptor (which also localizes along the ciliary axoneme) with *NfatC1^enCre^* ^,^ resulted in enlarged anterior valve leaflets with myxomatous MVP‐like phenotypes. PDGFRα suppresses EMT in a subset of valve endothelial cells by regulating the serine/threonine kinase (protein kinase B)/Extracellular signal‐regulated kinase (AKT/ERK) pathway that stabilizes the valve endocardium and prevents a disease phenotype (Moore et al., 2021).

### 6.2 Primary cilia and associated signaling defects in cardiac atria, ventricle, and other heart structures

AV septal defects (AVSDs) are CHDs commonly associated with heterotaxy syndrome (Francis et al., 2012; Icardo & Sanchez de Vega, 1991; Kathiriya & Srivastava, 2000; Kennedy et al., 2007; Seo et al., 1992; S. Y. Tan et al., 2007). AV septation and dorsal mesenchymal protrusion structure development have been attributed to the migration of second heart field (SHF) progenitor cells (Deepe et al., 2020). Cilia‐mediated Hh signaling is required for the SHF progenitor cell migration and OFT septation (Burnicka‐Turek et al., 2016; Goddeeris et al., 2008; Hoffmann et al., 2009; Washington Smoak et al., 2005). As previously mentioned, cilia‐regulated Hh signaling also plays an important role in laterality establishment (Hu et al., 2017), suggesting a link between AVSD and heterotaxy syndrome. This mechanistic link was further verified with the identification of the first human AVSD gene *CRELD1*, which is also a component of cilia (Beckert et al., 2021; Burnicka‐Turek et al., 2016).

Turek et al. (2016) identified recessive mutant alleles in two other cilia genes, *Dnah11* and *Mks1*. The *Dnah11* mutation results in AVSD with no disturbance in SHF Hh signaling (Burnicka‐Turek et al., 2016). *DNAH11* is an ODA component required for cilia motility, and disruption of its function is known to cause laterality defects (Bartoloni et al., 2002; Dougherty et al., 2016). The AVSD observed in *Dnah11* mutants could be the result of disturbances in early situs establishment events (Burnicka‐Turek et al., 2016). Additionally, *Mks1*, a component of the ciliary basal body, causes AVSD when mutated, with downregulation of SHF Hh expression, which is independent of laterality defects (Burnicka‐Turek et al., 2016). Another *Mks1* mutant recovered by Cui et al. (2011) also showed CHDs, polycystic kidneys, and randomization of left–right patterning (Cui et al., 2011). The tectonic protein Tctn2, which resides in the transition zone of cilia, interacts with Msk1 and regulates Hh signaling. The *Tctn2* knockout mouse also shows phenotypes characteristic of cilia‐mediated Hh defects including cleft palate, polydactyly, VSD, and right‐sided stomach placement (Sang et al., 2011). Another cilia gene, *Ftm* (also called *Rpgrip1l*), localizes at the base of cilia and regulates Hh signaling. *Ftm* mutant embryos show left–right asymmetry defects with randomized heart looping (Vierkotten et al., 2007). Later during heart development, *Ftm* null hearts show perimembranous VSDs along with muscular ventral septa defects, diminished ventricle wall thickness, and decreases in cilia length, which correlate with reduced cell proliferation. This was attributed to reduced sonic Hh (Shh) and Pdgfra signaling in the ventricles. The Ftm mutant atria and atrial septa showed no defects during heart development and no signs of altered Shh signaling. Since Hh signaling is associated with the development of the atrial septa but no atrial septa developmental defects were observed in *Ftm* mutants, it is proposed that there are different mechanisms by which Hh signaling regulates atrial and ventricular development. Because no cilia were observed on ventricle septa cells, the defective ventricular septa development in *Ftm* null mice suggests that the signal for ventricle septal development originates from cilia‐bearing cells in ventricle walls or that ventricular wall cell proliferation contributes to the formation of ventricular septa (Gerhardt et al., 2013).

Loss of function mutants of IFT genes also display cardiac malformations. For example, *Ift88* mutant mice show defects in cardiac OFT septation, ventricular trabeculation, AVSD, and cardiac cushion EMT, with no localization of the Hh pathway transcription factor Gli2 in cardiac cilia (Burns et al., 2019; Clement et al., 2009; Willaredt et al., 2012). In a previous study, Washington Smoak et al. (2005) reported that *Shh* deletion results in abnormal migration of neural crest cells, contributing to arch artery and OFT septation defects. Using an *Ift88* hypomorphic allele generated by *N*‐ethyl‐*N*‐nitrosourea (ENU) mutagenesis, Willaredt et al. (2012) identified no defects in the migration pattern of cardiac neural crest cells (CNCC) into the heart, but CNCC already migrated into the pharyngeal arches lacked cilia and displayed defective Shh and Bmp2/4 signaling, which are required for OFT septation. Mutant embryos for the gene *Kif3a*, which is essential for anterograde intraflagellar transport, also show laterality defects along with defective development of the endocardial cushions and compact myocardium (Slough et al., 2008). Recent studies by Peralta et al. (2020) have identified a cilia‐independent, non‐canonical, role of IFT complex B proteins (Ift88, Ift54, and Ift20) in modulating the Hippo pathway effector YAP1 and restricting proepicardial and myocardial tissue size during development (Peralta et al., 2020).

### 6.3 The mechanosensory role of primary cilia in heart development

Primary cilia are known to have a role as fluid shear stress sensor at node (McGrath et al., 2003) and in the kidney (Nauli et al., 2006; Xu et al., 2007, 2009); however, the exact mechanism by which biomechanical forces regulate gene expression is not fully understood. Yet, the mechanosensory function of primary cilia during development cannot be ignored (Djenoune et al., 2023; Katoh et al., 2023). Extensive tissue remodeling during heart development dramatically changes cardiac fluid shear stress patterns (Garoffolo & Pesce, 2019). In a correlative study of primary cilia distribution on endothelial and endocardium cells in chicken, Van der Heiden et al. (2006) revealed an inverse relation between primary cilia distribution and expression of the high shear stress marker *Klf2*. Endothelial cells have higher shear stress, resulting in higher expression of stress marker *Klf2* with a decreased presence of cilia; likewise in endocardial cells where shear stress is low, Klf2 is not detected, and primary cilia are present. The primary cilia on endothelial and endocardial cells sense shear stress forces and transmit them to the cytoskeleton, triggering a response. In theory, these shear stresses could potentially play a role in shaping the structural organization of the heart chambers (Van der Heiden et al., 2006).

The primary cilia gene *Pkd2*, responsible for encoding an integral membrane glycoprotein that bears resemblance to subunits of calcium channels, is recognized for its role as a mechanosensor in both blood vessels (MacKay et al., 2020) and at the node (Yoshiba et al., 2012). *Pkd2* mutant embryos retained the presence of primary cilia in the heart (Slough et al., 2008). G. Wu et al. (2000) demonstrated that *Pkd2* mutant mice die in utero with renal failure and cardiac septation defects (G. Wu et al., 2000). The mutant hearts showed decreased endocardial cushion cellularity and a thin compact myocardium, compared to stage‐matched wild‐type hearts (Slough et al., 2008). Boulter et al. (2001) described mice carrying a targeted mutation in the polycystin‐1 gene (Pkd1), which also encodes an integral membrane protein that localizes to the primary cilium and interacts with Pkd2. Pkd1 mutant mice showed AVSD with disorganization and thinning of the myocardial wall and double‐outlet right ventricle (Boulter et al., 2001). A recent study in zebrafish has also identified a synergistic role for the *pkd* genes (*pkd1*, *pkd2*, and *pkd1l1*) in blood flow‐driven valve development by repressing the expression of *klf2a* and *klf2b* (Juan et al., 2023). During cardiac trabeculation, cardiac contraction and hemodynamic forces exert mechanical stresses, which are detected by primary cilia on ventricular endocardial cells and decoded by the flow‐responsive transcription factor Klf2a. This leads to the activation of *notch1b‐efnb2a‐nrg1* pathway, which regulates the cross‐talk between the endocardium and myocardium during cardiac trabeculation. Using *ift88* morphant zebrafish, Samsa et al. (2015) noted that primary cilia on endocardial cells are required for notch1b activation. However, *notch1* activation is independent of ciliary Hh signaling but is required for functional primary cilia (Samsa et al., 2015). This suggests that primary cilia might serve as sensors of shear stress, and this function could contribute to cardiac valve and chamber development.

## 7 CILIOPATHIES, CHDs, AND CLINICAL RELEVANCE

CHDs are often found in patients with clinically recognized syndromes and developmental disorders affecting cilia function (Barisic et al., 2015; Elbedour et al., 1994; Engesaeth et al., 1993). For instance, in motile ciliopathies, also known as PCD, nearly half of the patients exhibit abnormal situs, and approximately 3.5%–6% manifest CHD as a part of their clinical profile (Best et al., 2019; Kennedy et al., 2007; Noone et al., 2004; Shapiro et al., 2014). Autosomal dominant polycystic kidney disease (ADPKD), a ciliopathy, often presents with significant heart‐related complications, contributing to increased morbidity (Rahman et al., 2009). Notably, around a quarter of ADPKD patients receive a diagnosis of MVP, in addition to other cardiac anomalies (Toomer et al., 2019). Dysregulations of ECM in both MVP and ADPKD impair molecular architecture and function, contributing to the disease phenotype (Toomer et al., 2019; Wilson et al., 1992). In Bardet–Biedl syndrome (BBS), another ciliopathy, individuals exhibit a 170‐fold higher prevalence of laterality defects, compared to the general population, although this occurrence remains lower than in patients with primary ciliary dyskinesia (PCD) (Olson et al., 2019). Furthermore, individuals with BBS also display defects such as AVSDs, vascular anomalies, and dilated cardiomyopathy (Niederlova et al., 2019; Yadav et al., 2013). Trisomy 21, a chromosomal copy number disorder, is associated not only with neurological abnormalities but also with CHD. Disruption of primary cilia formation and signaling in Trisomy 21 is attributed to elevated expression levels of the centrosomal protein Pericentrin, a gene located on chromosome 21 in humans. This excess Pericentrin disrupts ciliary protein trafficking and leads to defective Shh signaling as observed in murine Trisomy 21 models (Galati et al., 2018; Jewett et al., 2023). Pathogenic mutations in cilia gene *CRELD1* have also been identified in Trisomy 21 patients, resulting in AVSD defects (Asim et al., 2018).

To date, nearly 51 genes associated with PCD have been identified (Zhao et al., 2021). The majority of mutations fall into the category of loss‐of‐function variants (Knowles et al., 2013); however, copy number variants have also been detected in heterotaxy patients (Fakhro et al., 2011). Most of the affected PCD genes encode proteins associated with the cilia motility machinery (Y. Li et al., 2016; Reiter & Leroux, 2017). Nevertheless, alterations in genes that are active within the cytoplasm and participate in the preliminary formation of cilia have also been associated with PCD (Horani et al., 2012, 2013; Knowles et al., 2013). For instance, Nakhleh et al. (2012) observed that a notable percentage of individuals with heterotaxy and congenital heart disease exhibited impaired cilia function with an irregular ciliary beat pattern, although the ciliary structure itself remained intact. This phenomenon might signify a modified manifestation of PCD (Nakhleh et al., 2012).

## 8 CONCLUDING REMARKS AND PERSPECTIVES

Advances in molecular biology, disease modeling, microscopy and genome sequencing have illuminated the pivotal role of the minuscule cellular organelle, the cilium, in the establishment of cardiac asymmetry and heart function. Investigations employing patient‐specific cohort studies for CHD or forward genetic screens in mice have uncovered novel *de novo* mutations in cilia‐related genes. Furthermore, mutations in genes involved in pathways signaling through cilia localization have also emerged as contributors to cardiac developmental abnormalities. Experiments conducted in lower vertebrate animal models and human cell lines have further deepened our understanding of the multifaceted functions of cilia in development and homeostasis. The ongoing discovery of new cilia genes associated with CHD holds the promise for enhancing clinical awareness of the genetic causes of CHD, leading to guidance for family planning, improved prognosis, and the development of prenatal genetic screening tests for complex CHD cases requiring urgent intervention. Nonetheless, our knowledge regarding the precise involvement of cilia in regulating cardiac development remains in its nascent stages. Additional research is imperative to elucidate the diverse functions of motile and non‐motile primary cilia in cardiac development and function.

## AUTHOR CONTRIBUTIONS

Wasay Mohiuddin Shaikh Qureshi performed research and wrote the manuscript. Kathryn E. Hentges wrote the manuscript, edited the manuscript, and obtained funding.

## Data Availability

The data that support the findings of this study are openly available in Pubmed at https://pubmed.ncbi.nlm.nih.gov.
